# Characteristics and outcomes of patients infected with nCoV19 requiring invasive mechanical ventilation in Argentina

**DOI:** 10.5935/0103-507X.20200062

**Published:** 2020

**Authors:** Gustavo A. Plotnikow, Amelia Matesa, Juan M. Nadur, Marcelo Alonso, Ignacio Nuñez I, Gabriel Vergara, Maria J. Alfageme, Agustin Vitale, Marco Gil, Valeria Kinzler, Marianela Melia, Florencia Pugliese, Mariana Donnianni, Joana Pochettino, Ignacio Brozzi, Jose Luis Scapellato, Grupo Argentino Telegram nCoV19

**Affiliations:** 1 Sanatorio Anchorena - Buenos Aires, Argentina.; 2 Clínica Basilea - Buenos Aires, Argentina.; 3 Clínica de Internación Aguda en Rehabilitación y Cirugía - Buenos Aires, Argentina.; 4 Clínica Pasteur - Neuquén, Argentina.; 5 Hospital Evita Pueblo - Buenos Aires, Argentina.; 6 Sanatorio Femechaco de la Comunidad - Chaco, Argentina.; 7 Hospital San Miguel Arcángel - Buenos Aires, Argentina.; 8 Hospital Interzonal General de Agudos Eva Perón - Buenos Aires, Argentina.; 9 Hospital Dr. Francisco López Lima - Río Negro, Argentina.; 10 Hospital Dr Jaime Ferre - Santa Fe, Argentina.; 11 Hospital Comunitario de Pinamar - Buenos Aires, Argentina.; 12 Hospital General de Agudos Dr. D. Velez Sarsfield - Buenos Aires, Argentina.; 13 Hospital General de Agudos Bernardino Rivadavia - Buenos Aires, Argentina.; 14 Hospital Privado Universitario de Córdoba - Córdoba, Argentina.; 15 Grupo Argentino Telegram nCoV19 - Buenos Aires, Argentina.

**Keywords:** Respiration, artificial, Coronavirus, Coronavirus infections, COVID-19, Acute respiratory failure, Critical care, Respiración artificial, Infecciones por coronavirus, COVID-19, Coronavirus, Insuficiencia respiratoria aguda, Cuidados críticos

## Abstract

**Objective:**

A novel coronavirus emerged this year as a cause of viral pneumonia. The main characteristics of the virus are rapid transmission, high contagion capacity and potential severity. The objective of this case series study is to describe the clinical characteristics of patients with confirmed coronavirus disease (COVID-19) admitted to different intensive care units in Argentina for mechanical ventilation.

**Methods:**

A descriptive, prospective, multicenter case series study was conducted between April 1 and May 8, 2020. Data from patients older than 18 years who were admitted to the intensive care unit for mechanical ventilation for acute respiratory failure with a positive diagnosis of COVID-19 were included.

**Results:**

The variables for 47 patients from 31 intensive care units were recorded: 78.7% were men (median age of 61 years), with a SAPS II score of 43 and a Charlson index score of 3. The initial ventilatory mode was volume control - continuous mandatory ventilation with a tidal volume less than 8mL/kg in 100% of cases, with a median positive end-expiratory pressure of 10.5cmH_2_O. At the end of the study, 29 patients died, 8 were discharged, and 10 remained hospitalized. The SAPS II score was higher among patients who died (p = 0.046). Charlson comorbidity index was associated with higher mortality (OR = 2.27, 95% CI 1.13 - 4.55, p = 0.02).

**Conclusion:**

Patients with COVID-19 and on mechanical ventilation in this series presented clinical variables similar to those described to date in other international reports. Our findings provide data that may predict outcomes.

## Abstract (es)

**Objetivo:**

El coronavirus ha emergido este año como causa de neumonía viral. Una de las principales características es su rápida transmisión y su potencial severidad. El objetivo de este estudio de serie de casos es describir las características clínicas de los pacientes con confirmación de enfermedad por coronavirus (COVID-19) admitidos en diferentes unidades de cuidados intensivos de la Argentina con requerimiento de ventilación mecánica.

**Métodos:**

Estudio de serie de casos, descriptivo-prospectivo, multicéntrico realizado entre el 01 de abril y el 08 de mayo de 2020. Se incluyeron los datos de los pacientes mayores a 18 años, que ingresaron a la unidad de cuidados intensivos con requerimiento de ventilación mecánica por falla respiratoria aguda con diagnóstico positivo de COVID-19

**Resultados:**

Se registraron las variables de 47 pacientes de 31 unidades cuidados intensivos, 78.7% hombres de una mediana de edad de 61 años, con un SAPS II de 43, un índice de Charlson de 3. El modo ventilatorio inicial *fue volume control* - *continuous mandatory ventilation* con volumen corriente menor a 8mL/kg en el 100% de los casos, con una mediana de presión positiva al final de la espiración de 10,5cmH2O. A la fecha de cierre del estudio, 29 pacientes fallecieron, 8 alcanzaron el alta, y 10 pacientes continúan internados al cierre del estudio. El SAPS II fue mayor entre los fallecidos (p = 0.046). El índice de Charlson se asoció con mayor mortalidad (OR = 2,27 IC95% 1,13 - 4,55; p = 0,02).

**Conclusión:**

Los pacientes con COVID-19 y ventilación mecánica de esta serie presentan variables clínicas similares a las descriptas a la fecha en otros reportes internacionales. Nuestros hallazgos proporcionan datos que permitirían de alguna manera predecir los resultados.

## INTRODUCTION

A novel coronavirus (nCoV19) emerged this year as a cause of viral pneumonia. The main characteristics of the virus are rapid transmission, high contagion capacity and potential severity, which have resulted in the characterization of the infection as a pandemic by the World Health Organization (WHO).^(1,2)^

The high transmissibility of the virus and disease severity, often requiring admission to the intensive care unit (ICU) and mechanical ventilation (MV), oblige to reconsider all of the treatment standards known up to date. On May 8, 2020, in Argentina, the Ministry of Health reported a total of 5,371 people with a confirmed diagnosis of COVID-19 and a total of 155 inpatients in ICUs on that day.^(3)^ Among the patients hospitalized with COVID-19 worldwide, the percentage requiring ICU care has varied from 5% to 32%.^(4-8)^ The data on the incidence and clinical characteristics of critically ill patients diagnosed with COVID-19 are still limited. It is crucial to determine the admission characteristics and outcomes of critically ill patients requiring MV.

The objective of this case series study are to describe the clinical characteristics of patients requiring invasive MV with laboratory-confirmed COVID-19 admitted to different ICUs in Argentina and to determine predictors of ICU mortality.

## METHODS

This was a descriptive, prospective, multicenter (centers in Buenos Aires, Chaco, Santa Fe, Río Negro, Córdoba, Neuquén and the Autonomous City of Buenos Aires) case series study conducted between April 1 and May 8, 2020. The study was carried out by a multidisciplinary collaborative group composed of respiratory and physical therapists, physicians, and nurses, gathered through the Telegram network, which has 1,872 participants from all over the country.

A data collection form was created by the authors and then evaluated by 2 independent reviewers.

Patients older than 18 years who were admitted to the ICU and required MV for acute respiratory failure with a positive diagnosis of COVID-19 were included.

The clinical data reported in this study were prospectively collected through a digital form. The following data were collected: age, sex, anthropometric variables (height and weight), body mass index, comorbidities, Charlson comorbidity index, severity classification systems during the first 24 hours of admission (Simplified Acute Physiology Score II - SAPS II and Acute Physiology and Chronic Health Evaluation II - APACHE II), initial ventilatory support mode (invasive mechanical ventilation, noninvasive mechanical ventilation (NIMV), or high-flow oxygen therapy), initial programming variables (predicted body weight calculation, tidal volume (Vt) selection, and positive end-expiratory pressure (PEEP) selection strategy), ventilatory monitoring variables (peak pressure, plateau pressure, and total PEEP (PEEPt), driving pressure (DP), static compliance (Cst), initial arterial oxygen/fraction of inspired oxygen ratio (PaO_2_/FIO_2_) pressure and PaO_2_/FIO_2_ pressure on the day of extubation, use of rescue measures for hypoxia and hypoxemia (neuromuscular blockers, prone position, nitric oxide, and extracorporeal membrane oxygenation - ECMO), tracheostomy, number of days on MV, number of days in the ICU and number of days in the hospital. The outcome variables were survival and death.

### Statistical analysis

The analyses were performed by a statistician. A sample size calculation was not performed; the total number of patients treated during the study period was considered the sample size. Continuous data are expressed as the mean and standard deviation (SD) or as the median and interquartile range , according to their frequency distribution. Categorical data are expressed as absolute values and/or percentages. Variables were compared among patients using the Student’s t-test, Chi^2^ test or Mann-Whitney U test, according to the nature of the data. Independent risk factors for mortality were evaluated using logistic regression. A value of p < 0.05 was assumed to be significant. Logistic regression was used to evaluate independent predictors of mortality, which was the main outcome variable. The strength of an association is expressed as the odds ratio (OR) and 95% confidence interval (95%CI). SPSS version 20 (IBM Corp, Armonk, NY) was used for the statistical analyses.

The study was approved by the Teaching and Research Committee of *Sanatorio Anchorena Recoleta* under code F004-02-A (01) 2020. We dispensed with informed consent; however, the patient data were coded in such a way to achieve anonymity.

## RESULTS

The variables for 47 patients from 31 ICUs were recorded. The patients were mostly men (78.7%), with a median age of 61 (52.5 - 71) years, a median SAPS II score of 43 (31 - 64) points, an APACHE II score of 14 (7 - 20) points and a Charlson comorbidity index score of 3 (0 - 5) points. With respect to comorbidities, 36% of the patients had cardiovascular disease (peripheral vascular disease, infarction or congestive heart failure), 31% had hypertension, and 28% had diabetes (Table 1). No patient received NIMV, and only 1 received high-flow oxygen therapy.

**Table 1:** Description of the epidemiological variables in the total population and comparison between survivors and nonsurvivors

	Total N = 47	Nonsurvivors n = 29*	Survivors n = 8*	p value*
Age	61 (52.5 - 71)	66 (53 - 74)	52 (41 - 58.2)	0.051
Sex F/M	10/37	6/23	1/7	0.655
SAPS II score	43 (31 - 64)	62 (38 - 67)	24 (22 - 43)	0.046
APACHE II score	14 (7 - 20)	17 (8.5 - 23)	14 (7 - 18.5)	0.636
Charlson comorbidity index	3 (0 - 5)	4 (1 - 5)	0 (0 - 0.2)	0.02
Cardiovascular disease	17 (36.1)	15 (51.7)	0 (0)	0.08
Hypertension	13 (30.9)	10 (50)	2 (25)	0.44
Diabetes	13 (27.6)	9 (31)	2 (25)	0.74
COPD	5 (10.6)	3 (10.3)	1 (12.51)	0.86
Body mass index	28.4 (24.5 - 31.2)	27.7 (24.5 - 31.1)	30 (28 - 31.1)	0.22

SAPS II - Simplified Acute Physiology Score II; APACHE II - Acute Physiology and Chronic Health Evaluation II; COPD - chronic obstructive pulmonary disease.

* level of statistical significance between survivors and nonsurvivors (Chi^2^ test, Mann-Whitney U test, and t test as appropriate). Results expressed as median and interquartile range and n (%).

**Figure 1:**
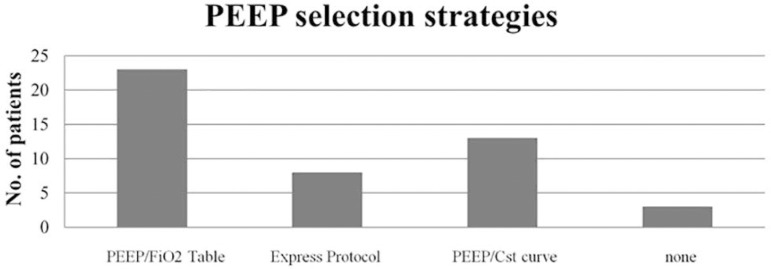
Positive end-expiratory pressure selection strategies distributed according to the number of patients in whom each of them was implemented. PEEP - positive end-expiratory pressure; Cst - static compliance.

The initial ventilatory mode in 100% of cases was volume control - continuous mandatory ventilation (VC-CMV). The predicted body weight was calculated for the selection of Vt in all cases and was between 6 - 8mL/kg for 76.5% of the patients and lower in the other patients. At baseline, patients had a median PaO_2_/FiO_2_ of 160 with a DP of 12cmH_2_O and a Cst of 39mL/cmH_2_O. Table 2 provides the MV and monitoring parameters. The median PEEP used was 10.5 (8.2 - 12) cmH_2_O, and a PEEP/FiO_2_ table was used in 48.9% of cases (Figure 1).

**Table 2:** Description of the outcome variables in the total population and comparisons between survivors and nonsurvivors

	Total N = 47	Nonsurvivors n = 29*	Survivors N = 8*	p value*
Peak pressure 1st day	30 (26 - 32)	30 (26.7 - 36)	27 (24.7 - 32)	0.273
Plateau pressure 1st day	22 (19 - 24)	22 (20 - 25.5)	19.5 (18 - 22.2)	0.154
PEEP (cmH2O) 1st day	10.5 (8.2 - 12)	11 (9.7 - 12)	10.5 (8 - 12.5)	0.723
Driving pressure (cmH_2_O) 1st day	12 (10 - 13)	12 (11 - 13)	10 (8.7 - 12.2)	0.164
Static compliance (mL/cmH_2_O) 1st day	39 (34.5 - 47)	37.5 (35 - 45)	44.2 (38.8 - 50)	0.420
PaO_2_/FiO_2_ 1st day	160 (127.5 - 196)	165.5 (138.7 - 189.5)	156 (124.2 - 245)	0.923
Use of neuromuscular blockers	45 (97.8)	25 (86.2)	8 (100)	0.557
Prone position	27 (61.3)	16 (61.5)	4 (50)	0.689
Extubation	10 (21.2)	1 (3.4)	7 (87.5)	< 0.000
PaO_2_/FiO_2_ extubation day	275 (208.2 - 284.5)	144 (118 - 156)	284 (277.5 - 300)	0.012
Tracheostomy	2 (4.2)	0 (0)	1 (12.5)	0.216
Weaned from MV	9 (19.1)	0 (0)	8 (100)	< 0.000
Duration of MV (days)	9 (5 - 15.5)	6 (5 - 9)	12 (7.7 - 16.2)	0.039
Length of ICU stay (days)	11 (6 - 18)	7 (5 - 10)	19 (14.5 - 23.5)	< 0.000
Length of hospital stay (days)	12 (6 - 21.2)	9 (5 - 12)	26 (22.5 - 27.5)	< 0.000

PEEP - positive end-expiratory pressure; PaO_2_/FiO_2_ - pressure of arterial oxygen/fraction of inspired oxygen ratio; IQR - interquartile range; MV - mechanical ventilation; ICU - intensive care unit.

* level of statistical significance between survivors and nonsurvivors (Chi^2^ test, Mann-Whitney U test, and t test as appropriate). Results expressed as median and interquartile range and n (%).

Of the 47 patients, 29 died (62%), 8 were discharged, and 10 were still hospitalized at the end of the study (1 extubated and 9 on MV) (Table 2). The SAPS II score was higher among deceased patients.

In the logistic regression, the only variable that was associated with higher mortality was the Charlson comorbidity index (OR = 2.27, 95%CI 1.13 - 4.55; p = 0.02) (Table 3).

**Table 3:** Variables evaluated in the logistic regression model

	B	OR	95%CI		p value
Charlson index	0.824	2.279	1.139	4.558	0.02
Age	0.047	1.052	0.997	1.112	0.064
SAPS II	0.085	1.088	0.994	1.191	0.066
APACHE II	0.031	1.031	0.933	1.14	0.548

OR - odds ratio; 95%CI - 95% confidence interval; SAPS II - Simplified Acute Physiology Score II; APACHE II - Acute Physiology and Chronic Health Evaluation II.

## DISCUSSION

In this case series of critically ill patients admitted to the ICU for MV with laboratory-confirmed COVID-19 in Argentina from April 1 to May 8, 2020, we found high mortality rate. Comorbidities, assessed by the Charlson index at admission, were an independent predictor of mortality.

The population in this study consisted mainly of older male adults (79%), which is similar to that reported in studies from Seattle^(6)^ and Lombardy,^(7)^ but higher than what was described in other studies.^(4,9,10)^ The median age of the patients admitted to the ICU was 61 years, which is higher than the median age of all patients positive for COVID-19 in Argentina.^(3)^ Although the variable age was associated with mortality in other studies, in our case series, it showed marginal value without a significant association, which may be explained by the low proportion of patients who survived until discharge.

Similar to our study, Yang et al.^(9)^ reported that the APACHE II score had failed to discriminate the severity of patients in relation to mortality, finding no differences between surviving and nonsurviving patients, and having values similar to those reported here (median APACHE II score day 1: 14/18, survivors/nonsurvivors, respectively). However, the SAPS II score did differ between groups, although it was not associated with higher mortality in the logistic regression.

In line with what was reported by Grasselli et al.^(7)^ (68%) and Wang et al.^(10)^ (72%), in this cohort of patients, 64% had at least 1 comorbidity, a higher percentage than that reported in other studies.^(4,5)^ Cardiovascular disorders were the most common comorbidities, followed by high blood pressure and diabetes, similar to other reports.^(4,10)^ The Charlson comorbidity index appears to be an interesting prognostic marker; however, no other study has documented this association with ICU mortality in a general population of patients infected with COVID-19.

At the onset of mechanical ventilation, patients presented gas exchange and pulmonary mechanic values similar to those described in previous cohorts.^(7,8)^ Subjects in this cohort were ventilated according to current guidelines for the management of patients with acute respiratory distress syndrome (ARDS), that is, ventilation with low Vt, moderate PEEP levels and low DP and plateau pressure.^(11)^ In addition, a greater use of neuromuscular blockers (97.8%) and prone decubitus (61.3%) was reported compared to the study by Ziehr et al.^(8)^ Unlike the studies by Bhatraju et al.^(6)^ and Ziehr et al.^(8)^ , the rate of extubation among the patients in this study was lower. However, although our cohort presents similarities in relation to the respiratory mechanics relative to the patients in those studies, it is not possible to compare the results because they did not any severity of admission.

Several studies reported different mortality rates among patients requiring admission to the ICU, from 16%^(10)^ to 78%.^(12)^ At the end of the present study, 21% of the patients were still in the ICU, 17% had been discharged from the ICU, and 62% had died in the ICU. It should be noted that the mortality rate reported in our study may be higher than in others because only patients who required MV were analyzed, and for this reason, they could have had a more severe baseline condition than those who did not require ventilatory support. Docherty et al.^(13)^ reported a similar mortality rate (53%) in a specific group of patients requiring MV. In addition, the elevated number of patients who required neuromuscular blockers and prone decubitus in our study could indicate higher mortality due to refractory hypoxemia, which has already been described for patients with ARDS.^(14)^ Similar to a study by Zhou et al.^(12)^ , who found that the risk of mortality during hospitalization increased with age (OR = 1.1, 95%CI 1.03 - 1.17, per year of increase; p = 0.0043), those who died in our cohort were the oldest.

This study has several limitations. First, although the data were recorded prospectively, the study design was retrospective. Second, the nature of the database did not allow obtaining more detailed information, such as ventilatory monitoring on days after the initial support or more specific laboratory data.

The number of cases was small; therefore, there may be independent determinants of mortality that could not be identified. It was also not possible to calculate the incidence of the disease because data regarding the populations of patients without COVID-19 who were admitted to the ICUs during the study period were not recorded. Lastly, the follow-up time was still relatively short compared to the disease course, and thus, the mortality and length of stay data could change.

## CONCLUSION

This study reports initial experiences regarding the clinical characteristics, respiratory parameters and mechanical conditions of the respiratory system of a group of patients infected with COVID-19 requiring mechanical ventilation admitted to different intensive care units in Argentina. Although further research is required to understand the impact of this disease, particularly in patients on mechanical ventilation, our findings provide data that would allow predicting the risk of mortality in affected patients.

## References

[1] Wax RS, Christian MD (2020). Practical recommendations for critical care and anesthesiology teams caring for novel coronavirus (2019-nCoV) patients. Can J Anaesth.

[2] World Health Organization Coronavirus disease (COVID-19) outbreak.

[3] Argentina, Ministerio de Salud Nuevo coronavirus COVID-19. Reporte Diario Matutino/08-05-2020.

[4] Guan WJ, Ni ZY, Hu Y, Liang WH, Ou CQ, He JX, Liu L, Shan H, Lei CL, Hui DSC, Du B, Li LJ, Zeng G, Yuen KY, Chen RC, Tang CL, Wang T, Chen PY, Xiang J, Li SY, Wang JL, Liang ZJ, Peng YX, Wei L, Liu Y, Hu YH, Peng P, Wang JM, Liu JY, Chen Z, Li G, Zheng ZJ, Qiu SQ, Luo J, Ye CJ, Zhu SY, Zhong NS, China Medical Treatment Expert Group for Covid-19 (2020). Clinical characteristics of coronavirus disease 2019 in China. N Engl J Med.

[5] Huang C, Wang Y, Li X, Ren L, Zhao J, Hu Y (2020). Clinical features of patients infected with 2019 novel coronavirus in Wuhan, China. Lancet.

[6] Bhatraju PK, Ghassemieh BJ, Nichols M, Kim R, Jerome KR, Nalla AK (2020). Covid-19 in critically ill patients in the Seattle Region-case series. N Engl J Med.

[7] Grasselli G, Zangrillo A, Zanella A, Antonelli M, Cabrini L, Castelli A (2020). Baseline characteristics and outcomes of 1591 patients infected with SARS-CoV-2 admitted to ICUs of the Lombardy Region, Italy. JAMA.

[8] Ziehr DR, Alladina J, Petri CR, Maley JH, Moskowitz A, Medoff BD (2020). Respiratory pathophysiology of mechanically ventilated patients with COVID-19: a cohort study. Am J Respir Crit Care Med.

[9] Yang X, Yu Y, Xu J, Shu H, Xia J, Liu H (2020). Clinical course and outcomes of critically ill patients with SARS-CoV-2 pneumonia in Wuhan, China: a single-centered, retrospective, observational study. Lancet Respir Med.

[10] Wang D, Hu B, Hu C, Zhu F, Liu X, Zhang J (2020). Clinical characteristics of 138 hospitalized patients with 2019 novel coronavirus-infected pneumonia in Wuhan, China. JAMA.

[11] Fan E, Del Sorbo L, Goligher EC, Hodgson CL, Munshi L, Walkey AJ, Adhikari NKJ, Amato MBP, Branson R, Brower RG, Ferguson ND, Gajic O, Gattinoni L, Hess D, Mancebo J, Meade MO, McAuley DF, Pesenti A, Ranieri VM, Rubenfeld GD, Rubin E, Seckel M, Slutsky AS, Talmor D, Thompson BT, Wunsch H, Uleryk E, Brozek J, Brochard LJ, American Thoracic Society, European Society of Intensive Care Medicine, and Society of Critical Care Medicine (2017). An Official American Thoracic Society/European Society of Intensive Care Medicine/Society of Critical Care Medicine Clinical Practice Guideline: Mechanical Ventilation in Adult Patients with Acute Respiratory Distress Syndrome. Am J Respir Crit Care Med.

[12] Zhou F, Yu T, Du R, Fan G, Liu Y, Liu Z (2020). Clinical course and risk factors for mortality of adult inpatients with COVID-19 in Wuhan, China: a retrospective cohort study. Lancet.

[13] Docherty AB, Harrison EM, Green CA, Hardwick HE, Pius R, Norman L, Holden KA, Read JM, Dondelinger F, Carson G, Merson L, Lee J, Plotkin D, Sigfrid L, Halpin S, Jackson C, Gamble C, Horby PW, Nguyen-Van-Tam JS, Ho A, Russell CD, Dunning J, Openshaw PJ, Baillie JK, Semple MG, ISARIC4C investigators (2020). Features of 20,133 UK patients in hospital with COVID-19 using the ISARIC WHO Clinical Characterisation Protocol: prospective observational cohort study. BMJ.

[14] Estenssoro E, Dubin A, Laffaire E, Canales H, Sáenz G, Moseinco M (2002). Incidence, clinical course, and outcome in 217 patients with acute respiratory distress syndrome. Crit Care Med.

